# Grape Pomace: Agrifood By-Product with Potential to Enhance Performance, Yolk Quality, Antioxidant Capacity, and Eggshell Ultrastructure in Laying Hens

**DOI:** 10.3390/vetsci10070461

**Published:** 2023-07-13

**Authors:** Shaimaa Selim, Nazema S. Abdel-Megeid, Rashed A. Alhotan, Alia Ebrahim, Eman Hussein

**Affiliations:** 1 Department of Nutrition and Clinical Nutrition, Faculty of Veterinary Medicine, Menoufia University, Shibin El-Kom 32514, Egypt; 2 Department of Cytology and Histology, Faculty of Veterinary Medicine, University of Sadat City, Sadat City 32897, Egypt; 3 Department of Animal Production, College of Food and Agriculture Sciences, King Saud University, Riyadh 11451, Saudi Arabia; 4 Jiangsu Key Laboratory for Microbes and Genomics, School of Life Sciences, Nanjing Normal University, Nanjing 210098, China; 5 Department of Poultry and Fish Production, Faculty of Agriculture, University of Menoufia, Shibin El-Kom 32514, Egypt

**Keywords:** laying hens, grape pomace, laying rate, yolk fatty acid composition, oxidative stability, blood biochemistry, eggshell, tibia

## Abstract

**Simple Summary:**

Grape pomace is an agrifood by-product that is usually thrown away as a waste product, even with its rich content of phytochemicals. The aims of this study were to investigate the effects of dietary inclusion of grape pomace on laying rate, egg quality, yolk lipid profile, oxidative stability, shell quality and ultrastructure, and serum biochemistry in laying hens. Egg production, egg weight, and egg mass were improved due to the addition of grape pomace to the diets of laying hens. A reduction in saturated fatty acids was observed, while monounsaturated fatty acids, polyunsaturated fatty acids, and n-3 fatty acids were increased in the egg yolk of hens fed grape pomace. The dietary inclusion of grape pomace mitigated lipid oxidation and improved the antioxidant capacity of the fresh and stored eggs. The inclusion of grape pomace increased shell weight, thickness, and breaking strength by augmenting the thickness of the palisade layer. To conclude, the dietary inclusion of grape pomace in the diets of layers improved laying performance, enriched the yolk with beneficial fatty acids, augmented the antioxidant potential of yolk lipids, and enhanced shell quality and ultrastructure.

**Abstract:**

Grape pomace (GP) is an industrial by-product of grape juice making and is commonly discarded as a waste product, even with its large quantity of phytochemicals. Thus, the objectives of this trial were to examine the effects of graded dietary GP on laying rate, egg quality, yolk lipid profile, oxidative stability, shell quality and ultrastructure, and serum biochemistry. Two hundred 35-week-old laying hens were allocated to four dietary treatments with ten replicates each. Four diets were formulated by mixing a standard basal diet with GP at 0 g/kg (control), 30 g/kg (GP_3%_), 60 g/kg (GP_6%_), and 90 g/kg (GP_9%_). Egg production percent, egg weight, and egg mass were linearly improved (*p* < 0.01) due to adding GP to the diets of laying hens. Eggs obtained from laying hens fed with GP diets had (*p* < 0.01; linear, *p* < 0.01) greater Haugh units, yolk color, albumen index, and yolk index than those of the control. The GP_9%_ group had the greatest values (*p* < 0.05) for shell weight, thickness, and breaking strength. Electron microscopy scanning of eggshells indicated that the incremental dietary level of GP linearly augmented the thickness of the palisade layer but reduced both the mammillary layer and mammillary knob width (*p* < 0.01). Improved tibia-breaking strength and ash content were shown (*p* < 0.05) in the GP-fed laying hens. The dietary addition of GP by up to 90 g/kg linearly (*p* < 0.01) mitigated lipid oxidation and improved the antioxidant capacity in both the serum and stored eggs. A reduction in the percentages of saturated fatty acids was observed, while the contents of monounsaturated fatty acids, polyunsaturated fatty acids, and n-3 fatty acids were augmented because of increasing dietary GP levels (*p* < 0.001). Additionally, the eggs obtained from laying hens fed on the GP_6%_ and GP_9%_ had lower yolk cholesterol content (*p* < 0.001); this effect was confirmed by linear and quadratic responses (*p* < 0.001). Laying hens on GP diets had lower (*p* < 0.01) serum hepatic enzymes, cholesterol, triglycerides, and low-density lipoprotein but greater high-density lipoprotein compared to the control. To sum up, the addition of GP in the layers’ diets by up to 90 g/kg increased laying performance, enriched the yolk with beneficial fatty acids, enhanced antioxidant potential in yolk lipids, and improved shell quality and ultrastructure.

## 1. Introduction

Over the last decade, the continual growth of the human population has raised the requirement for poultry products worldwide, including meat and eggs [1]. Human consumption of hen eggs is progressively rising worldwide, with a rise of 14.1% per capita from 2010 to 2019 [2]. At the same time, the cost of feed ingredients, particularly protein sources and cereal grains, has increased. Feed ingredients present the greatest percentage of animal production costs, and thus observing sustainable and cost-effective practical alternative feedstuffs to replace traditional feedstuffs is very imperative [3]. Agro-industrial by-products are an extraordinary substitution option because these products are produced in considerable quantities yearly, predominantly from the juice and winery industries, causing a great challenge for waste disposal and management [3,4]. Likewise, with their accessibility and low price, these by-products are a valuable source of nutritional components such as fiber, minerals, vitamins, polyphenols, and flavonoids.

Eggs are a useful functional food owing to their constituents of essential amino acids, minerals, vitamins, and bioactive components [5,6,7]. Accordingly, adequate egg intake has been proven to induce health benefits in humans. Still, the nutritional value of the eggs can be improved by enriching them with polyunsaturated fatty acids (PUFA), which have been proven to be associated with a lower prevalence of human health-metabolic problems [8,9,10]. However, the eggs enriched with PUFA are more susceptible to lipid oxidation and deterioration, subsequently shortening the shelf-life of the eggs [11,12]. Lipid oxidation in eggs has conventionally been mitigated with the addition of antioxidant supplements, for instance vitamin E and canthaxanthin, to the diets of laying hens [11]. Despite this, natural alternative components with antioxidant activities can be added to the diets of laying hens to augment the egg’s oxidative stability, such as phenolic and flavonoid compounds [12]. However, the application of these extracted components from natural phytogenic plants is believed to be impractical because of the expensive extraction protocols.

Grape pomace (GP) is an industrial by-product of grape juice making and is commonly thrown away in landfills as a waste product, even with its large quantity of phytochemicals [13]. GP disposal can induce environmental pollution; accordingly, unconventional utilizations of GP are required to smooth the appropriate management and use of this by-product [14]. GP consisted of seedless pomace (residual pulp, stem, and skin, approximately 48–62%) and seeds (about 38–52%) [13]. GP is a good source of fundamental bioactive compounds with antioxidant, antibacterial, growth-stimulant, and improved meat quality [5]. Indeed, polyphenols present in grapes, particularly catechins, resveratrol, luteolin, quercetin, kaempferol, and anthocyanins, have been recognized to mitigate the scavenging capacity of free radicals and inhibit oxidation activities [13]. Consequently, its inclusion in poultry diets can beneficially lessen waste disposal, while generating healthier poultry products. Nevertheless, the GP contents of tannins and structural carbohydrates may hinder their nutrient digestibility and utilization by birds [15,16]. Previous studies have shown that dietary supplementation with grape by-products, such as GP, seeds, and grape skins, was efficient in decreasing the meat’s lipid peroxidation without impairing growth performance in broiler chickens [13,15,16,17]. As far as we know, few studies are currently available about the dietary supplementation of GP in layers and its impact on egg quality traits [11,18,19,20]. These research reports revealed that GPs recommended dietary inclusion rate in poultry ranges from 15 to 60 g/kg [13,15,17,18,19,20].

To our knowledge, there is a scarcity of research assessing the effect of graded dietary GP on laying rate, yolk lipid peroxidation, fatty acid (FA) profile, shell ultrastructure, and egg shelf-life in laying hens. Therefore, we aimed to explore the effects of graded inclusion of GP in the diets of laying hens on laying performance, egg quality traits, egg yolk lipid oxidation, yolk FA profile, shell quality and ultrastructure, and egg shelf-life. These aims can attribute importance to saving conventional feedstuffs, enriching the research on the nutrition of laying hens, bridging the research gap in GP in poultry, and encouraging the advancement of an environmentally friendly economy. Our hypothesis was that the dietary inclusion of GP could modify the laying rate, egg quality, antioxidant capacity, and shell ultrastructure.

## 2. Materials and Methods

The current trial was performed under a protocol accepted by the Institutional Animal Care and Use Committee with oversight by the Faculty of Veterinary Medicine, Sadat City University, Egypt. The ethical approval number is VUSC-021-1-23.

### 2.1. Grape Pomace Analysis

GP originating from red grapes (*Vitis vinifera*) was collected from the Paste and Juice Company (P & J), Sadat City, Egypt. The proximate chemical composition of GP was performed following AOAC protocols [21]. Dietary phytate phosphorus was measured [22], and available phosphorus was calculated as total phosphorus minus phytate phosphorus. The metabolizable energy of GP has been obtained from the Egyptian Central Laboratory for Food and Feed (Tables of feed composition 2001, Bulletin no. 1, 8). Neutral detergent fiber (NDF) and acid detergent fiber (ADF) in GP were measured as described by Van-Soest et al. [23]. Total phenolic content was determined by the Folin-Ciocalteau reagent as described by Al-Farsi et al. [24]. Total flavonoid content was performed according to the methods of Kim et al. [25]. Phenolic acids in GP were measured by high-performance liquid chromatographic (HPLC) using the method of Mattila et al. [26]. The proximate analysis, total phenolic, total flavonoid, phenolic acids, and FA profiles of GP are shown in Table 1.

### 2.2. Trial Design, Diets, and Bird Management

The current study consisted of an eight-week trial designed with four dietary treatments with 10 replicates each in a completely randomized design. Two hundred 35-week-old Lohman Brown Lite laying hens with an initial body weight of 1823 ± 30 g were used. Laying hens were retained in cages (0.44 m × 0.30 m × 0.45 m) offered with nipple drinkers and trough feeders. The birds were vaccinated and managed according to the breeder standards. The light schedule was 16 h light and 8 h darkness daily, and the temperature in the laying hens’ house was 24 ± 3 °C during the trial. Feed, which was available in mash form, and water were kept on an ad libitum basis during the trial period. The dietary treatments were as follows: (1) the control diet (no GP, control); (2) a basal diet containing 3% GP (GP_3%_); (3) a basal diet containing 6% GP (GP_6%_); and (4) a basal diet containing 9% GP (GP_9%_). Diets were prepared to be isocaloric and isonitrogenous and to meet the nutrient requirements of the breeders. Experimental diets are shown in Table 2.

### 2.3. Production Performance and Egg Quality

The initial body weight of laying hens before the start of the experiment (35 weeks of age) and the final body weight (43 weeks of age) were recorded. Feed intake (FI) by hens was recorded weekly and then divided by the number of hens per replicate and by 7 to report the daily FI per laying hen. The laying rate and egg weights were determined weekly during the experiment. Egg mass was estimated by multiplying egg weight with laying rate, and the feed conversion ratio (FCR) was documented as g of FI divided by g of egg mass. Egg quality parameters, including albumen and yolk weights, albumen height, yolk height, Haugh unit, specific gravity, shell thickness, and yolk color score, were determined in 60 eggs obtained from each replicate during the last 3 days of week 8 of the experiment. Shell thickness was assessed by micrometer after the removal of shell membranes, and the average value of 3 locations on the egg (at the 2 poles and the middle) was documented. Albumen and yolk heights were measured by a standard tripod micrometer, and the yolk diameter was determined by a caliper. Egg shape index (%), egg yolk index (%), and egg yolk albumen index (%) were accomplished by determining egg width and length using a caliper. Measuring the egg yolk color of freshly laid eggs was performed by the Roche yolk color fan. Haugh units were calculated using the formula described in [27].

### 2.4. Egg Yolk Lipid Profile, Oxidation Stability, and Fatty Acid Composition

On the 2nd day of the last week of the experiment (43 weeks old), 30 freshly laid eggs were obtained from each group (a total of 120 eggs) to assess the egg yolk cholesterol and triglyceride concentrations. The eggs were broken, and the yolks were pooled and separated from the egg white. Yolk cholesterol (mg/g) and triglyceride (mg/g) contents were determined spectrophotometrically using an ultraviolet spectrophotometer UV4802 (Unico Co., Dayton, OH, USA) and commercial kits (Sigma-Aldrich, St. Louis, MO, USA) based on the methods of Hammad et al. [28] and Kaya et al. [29]. On the 3rd day of the last week of the experiment, 10 freshly laid eggs were gathered from each group. The MDA was estimated on the day of egg collection. Moreover, another 30 freshly laid eggs were obtained per group and stored at 4 °C for 25 and 45 days. After the storage time, these eggs were broken and the yolks pooled, then MDA and GPx values were measured to evaluate the degree of yolk lipid peroxidation according to Botsoglou et al. [30], Galobart et al. [31], and Paglia and Valentine [32]. On the 4th day of the last week of the study, 20 freshly laid eggs were obtained from each group and broken, and the yolk was separated from the egg albumen. Then, the collected yolks were pooled and then frozen at −20 °C, freeze-dried, and later used for the measurement of the yolk FA composition using the protocol explained by Yang et al. [33] with a gas chromatograph (Model GC-14A, Shimadzu Corporation, Kyoto, Japan). The FA percentage was calculated as a percent of the total FA in the egg yolk.

### 2.5. Tibia Physical Traits and Chemical Composition

To determine the tibia bone, 10 hens from each group (1 bird/replicate) with the same homogeneity were chosen and euthanized at the end of the trial to gather tibia bones. Meat and other adhering tissue were removed from the tibia. The weight of the tibia (g) was determined by a decimal digital scale, and the tibia’s length and width (mm) were measured by a digital micrometer. Tibia-breaking strength was performed and expressed as kilograms’ force according to the method of Flemming et al. [34]. The tibia bones were crushed and dried in a hot air oven for 24 h at 105 °C. The samples were defatted by the Soxhlet method using petroleum ether and then dried at 100 °C for 24 h. The dried fat-free tibia was ignited in a muffle furnace at 600 °C overnight, and tibia ash content was represented as a percent of the fat-free dry basis.

### 2.6. Electron Microscope Scanning of Eggshell Ultrastructure

At the end of week 8 of the trial, one egg was randomly collected from each replicate (10 eggs/treatment). Before SEM imaging, the eggs were broken, then the eggshells were washed with distilled water to remove any dirt and dried at room temperature for 48 h [34]. Samples of eggshells (0.5–1 cm^2^) were arranged for scanning electron microscope analysis. One sample was used to assess the cross-section of the eggshell, and the other was set to evaluate the external surface. Samples were mounted on aluminum stubs, coated with gold in an SPI-Module ™ Vac/Sputter, and then photographed using an electron microscope (JEOL, JSM- 5200 LV scanning electron microscope, Japan) at an electron microscope unit at Tanta University, Egypt. The photos were taken to analyze the cross-section of the eggshell at ×100 magnification, and the others were used to assess the external surface of the eggshell at ×35 magnification. The palisade layer thickness (µm), mammillary layer thickness (µm), and total thickness (palisade and mammillary, µm) were determined following Stefanello et al. [35] using Image J software version 1.53t.

### 2.7. Serum Biochemistry

At the end of the trial (43 weeks of age), blood samples were obtained from the wing vein of laying hens (20 samples per treatment) for assessing serum metabolites. Serum was taken by centrifugation at 3000 rpm for 5 min and stored at −20 °C till further analysis. The serum samples were analyzed spectrophotometrically (spectrophotometer UV4802, Unico Co., Dayton, OH, USA) using commercial kits (Bio-diagnostic Co., Cairo, Egypt) for measuring total protein, albumin, total lipids, triglycerides, cholesterol, aspartate aminotransferase (AST), and alanine aminotransferase (ALT) levels. Serum MDA [36] and GPx [37] levels were measured spectrophotometrically using commercial kits (Bio-diagnostic Co., Cairo, Egypt).

### 2.8. Statistical Analysis

The normality of the data was checked by Kolmogorov-Smirnov and Levene’s tests. Data were analyzed by One-way ANOVA using IBM SPSS software version 21, and Tukey’s test determined significant variations between the treatment groups (*p* < 0.05). The experimental unit was the replicate for production data and the bird for other measurements. Furthermore, the incremental levels of GP in the diets of laying hens were assessed by orthogonal polynomial contrasts, and the *p*-value was documented. All values are presented as mean ± SEM.

## 3. Results

### 3.1. Laying Performance

The initial body weight (BW) and final BW of laying hens did not differ among the treatment groups (Table 3). Egg production percent during the study period (from week 35 to week 43 of age) was significantly enhanced (*p* = 0.007) due to feeding GP in layers’ diets; this pattern was confirmed by a linear response (*p* < 0.01) (Table 3). In hens fed the GP diets, there were significant increases (*p* < 0.01) in egg weight and egg mass compared to those fed the control diets; this effect was indicated by a linear response only (*p* < 0.001) (Table 3). The largest egg weight and egg mass were observed in the laying hens fed the GP_9%_ diet. Hens fed the GP_6%_ and GP_9%_ had lower FI (*p* < 0.01; linear, *p* < 0.001; quadratic, *p* < 0.05) during the trial period (from week 35 to week 43 of age) compared to the control (Table 3). On the contrary, the feed conversion ratio (FCR) was enhanced (*p* < 0.001) in all the GP-fed groups compared to the control; this response was implied by linear (*p* < 0.001) and quadratic (*p* < 0.05) responses (Table 3).

### 3.2. Egg Yolk and Albumen Quality

Dietary modification with GP resulted in greater yolk color scores (*p* < 0.001; linear, *p* < 0.001; quadratic, *p* < 0.01; cubic, *p* < 0.05) concerning the values recorded in the eggs of the control hens (Table 4). The GP-fed hens had a lower yolk weight % (*p* < 0.01) but a higher albumen weight % (*p* < 0.01) than the control hens (Table 4). As recorded in Table 4, the indexes of egg yolk and albumen were significantly increased (*p* ˂ 0.001) in the GP-fed hens as compared to the control ones. The dietary inclusion of GP resulted in a linear increase (*p* < 0.01) in the Haugh unit, particularly at 90 g/kg, as compared with the control group (Table 4). However, a non-significant effect (*p* > 0.05) was noticed among treatments for the yolk and albumen pH values (Table 4).

### 3.3. Egg Yolk Lipid Profile

Table 5 shows that egg yolk SFA contents linearly (*p* < 0.001) and quadratically (*p* < 0.05) decreased in response to GP levels. Laying hens on the GP_9%_ diets had the lowest yolk SFA contents compared to other treatment groups. On the other hand, laying hens on the control diets had lower egg yolk MUFA (*p* < 0.001) and PUFA (*p* < 0.001) concentrations compared to those on the GP diets (Table 5). Similarly, control diets prompted lower (*p* < 0.001) n-6 FA than GP diets (linear, *p* < 0.001, quadratic, *p* < 0.01; cubic, *p* < 0.001). Laying hens on the GP diets had greater egg yolk n-3 FA contents compared to those on the control diets; this effect was supported by linear, quadratic, and cubic responses (*p* < 0.05). The GP_9%_ group prompted the greatest level of yolk n-3 FA and the lowest n6:n3 FA ratio compared to the other treatment groups (Table 5). Dietary inclusion of GP linearly, quadratically, and cubically (*p* < 0.05) decreased the ratio of SFA to either PUFA or MUFA (control vs. GP, *p*< 0.001) in the egg yolk compared to the control diet. Furthermore, the eggs obtained from laying hens fed on the GP_6%_ and GP_9%_ had lower yolk cholesterol concentrations (*p* < 0.001) compared to those obtained from the control and GP_3%_ groups; this response was confirmed by linear and quadratic effects (*p* < 0.001). Eggs obtained from all GP-laying hens had a lower yolk triglyceride concentration (*p* < 0.001) (Table 5).

### 3.4. Egg Yolk Oxidative Stability

Table 6 presents the effect of the experimental diets on yolk oxidative stability during storage. There were significant dietary effects on GPx, where freshly laid eggs from laying hens in diet GP_6%_ and GP_9%_ had greater yolk GPx contents (*p* < 0.001) than those from diet control. However, the control diet promoted a similar (*p* > 0.05) yolk GPx level as diet GP_3%_. Similarly, after 25 and 45 days of storage, the eggs from the GP_6%_ and GP_9%_ groups had higher (*p* < 0.01) egg yolk GPx contents; this effect was characterized by a linear response (*p* < 0.01). Regarding the yolk MDA concentrations of freshly laid eggs, there were non-significant differences among the experimental groups. However, after storage for 25 days, the GP_6%_ and GP_9%_ eggs had lower yolk MDA levels (*p* < 0.001; linear, *p* < 0.001) when compared to the control and GP_3%_ ones. For extended storage duration (45 days), all GP groups prompted lower levels of MDA in egg yolk (*p* < 0.001; linear, *p* < 0.001; quadratic, *p* < 0.01) compared to the control group.

### 3.5. Tibia Physical Traits and Chemical Composition

Data on tibia physical morphology and chemical composition are presented in Table 7. Dietary modification with GP significantly did not induce any variations in the weight, length, or width of the tibia. Tibia bone dry matter % did not differ among the four treatments. The present results showed that the tibia bone-breaking strength (*p* < 0.001) and ash percent (*p* < 0.05) significantly increased and correlated with increasing GP levels (linear, *p* < 0.01).

### 3.6. Shell Quality and Ultrastructure (Electron Microscope)

**Figure 1:**
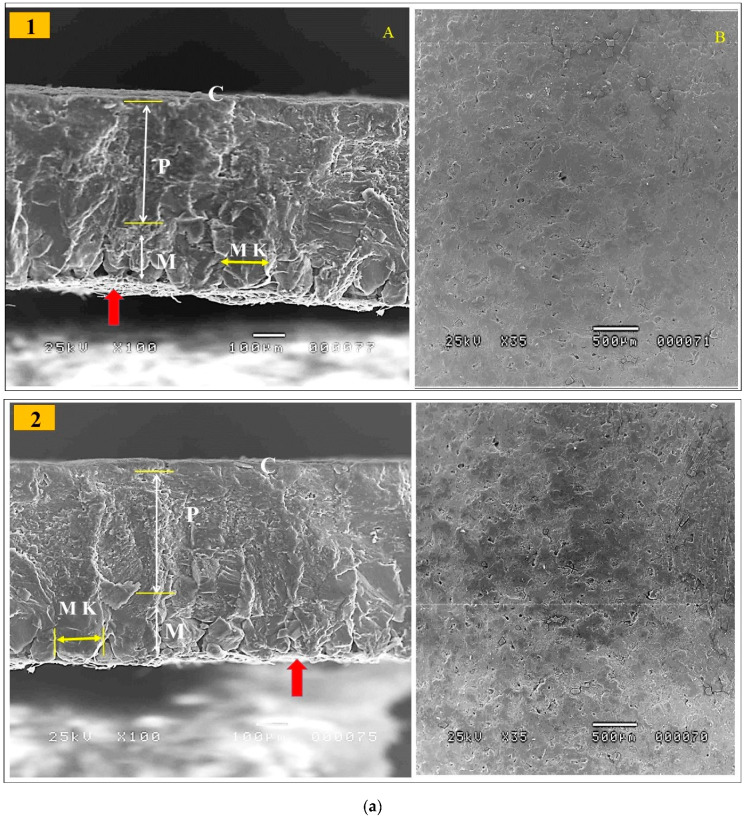

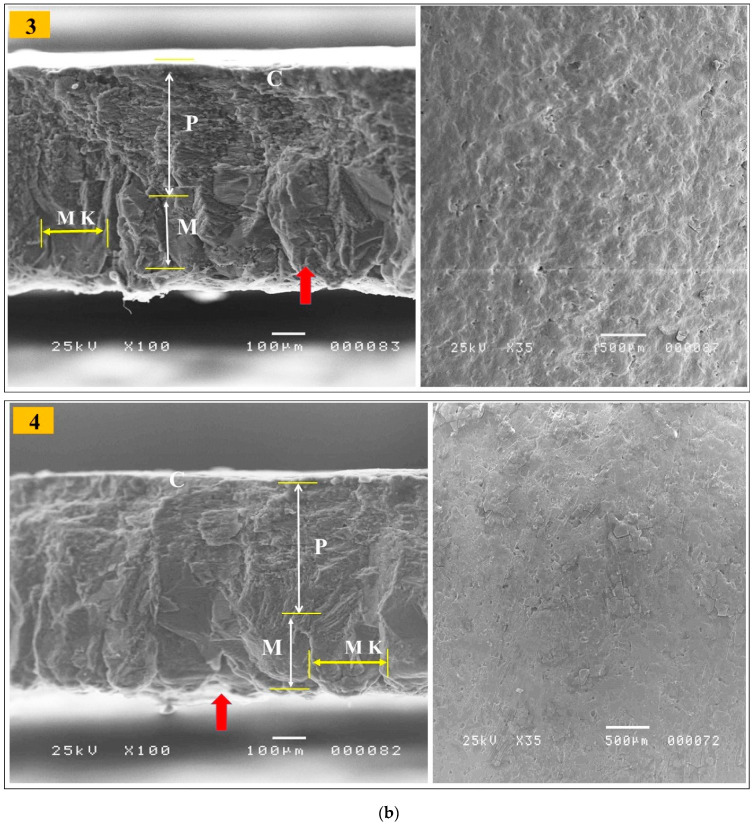
(**a**,**b**) Electron microscopy scanning of the eggshell cross-section (**A**) and the external surface (**B**) of laying hens fed the control diet (**1**), the GP3% (**2**), the GP_6%_ (**3**) and the GP_9%_ (**4**) diets at week 43 of age. Cuticle (C), palisade layer (P), mammillary layer (M), mammillary knobs (MK), and white-shell side membrane fibers (red arrow). For eggshell ultrastructure, see Section 3.6.

Table 8 shows that shell weight percentage, thickness, and breaking strength were linearly improved (*p* < 0.01) in response to the increased dietary levels of GP. The GP_9%_ group had the greatest values for shell weight, thickness, and breaking strength (Table 8). Data on the eggshell ultrastructure of laying hens at the end of the trial (43 weeks of age) by scanning electron microscopy are presented in Table 8 and Figure 1a,b. The eggshell is a smooth, hard, calcareous structure that is firmly attached to the outer shell membrane. The mineral crystals on the surface form many tiny pores in the bird’s eggshell. The eggshell is formed from an inner layer called the testa and an outer layer called the cuticle. The testa is composed of an organic network of delicate protein fibers and an inorganic interstitial substance of inorganic salts, mainly calcium carbonate. The cuticle is an organic outer layer of eggshell that is composed of a protein matrix lined with mineral crystals, usually calcium carbonate. Most of the eggshell is composed of calcium carbonate crystals (approximately 95%). The mammillary layer is a thin layer of spherolithic calcite crystal aggregates acting as overturned cones, which join to make a compact palisade layer. The palisade layer is the thickest part of the eggshell and combines with the outermost crystalline layer, which is protected by a cuticle. The cuticle contains hydroxyapatite crystals and most of the pigment. The entire eggshell is porous, and pore channels appear between the mammillary cones, spreading radially across the palisade and reaching the exterior. Thus, these channels penetrate all levels of the eggshell, enabling the exchange of water and metabolic gases. The incremental level of GP had linear and quadratic effects (*p* < 0.01) on the palisade layer thickness compared to the control; the highest thickness was observed in the GP9% eggshell. There was non-significant variation in the mammillary layer thickness among the experimental group (Figure 1). Lower mammillary thickness % was observed in the GP_9%_ compared to other treatment groups (*p* < 0.01). Lower mammillary knob width was recognized in the GP-added groups, particularly GP_6%_ and GP_9%_, compared to the control; this effect was characterized by a linear response (*p* < 0.01).

### 3.7. Serum Biochemistry

Data on serum biochemical constituents are shown in Table 9. Serum total protein and albumin concentrations were linearly (*p* < 0.001) and cubically (*p* < 0.05) increased in the GP-fed groups compared to the control ones; the effect was more pronounced for the GP_6%_ and GP_9%_ hens (*p* < 0.001). There was a non-significant difference in serum globulin content among the treatment groups. Laying hens fed the GP diets had lower (*p* < 0.01) serum total cholesterol, triglycerides, and LDL levels, particularly in the GP_6%_ and GP_9%_ hens, than the control (linear, *p* < 0.001). In contrast, the GP-fed hens had a higher serum HDL concentration than the control group (linear, quadratic, and cubic, *p* < 0.001). The ALT and AST concentrations were linearly decreased in the serum of laying hens fed the GP diets when compared to those fed the control diet. The GP-fed hens had significantly greater serum levels of GPx (*p* < 0.001; linear, *p* < 0.001; cubic, *p* < 0.001) and lower levels of MDA (*p* < 0.001; linear, *p* < 0.001) than the control hens.

## 4. Discussion

Grape pomace can possibly be used as a practical feedstuff in poultry nutrition owing to its rich bioactive components (phenolic acids and flavonoids) with favorable antioxidant and antimicrobial activities [13,15]. Nonetheless, the structural carbohydrates and phenolic components present in GP may constrain its utilization in poultry diets [11,20,38]. At the same time, there is a dearth of research investigating the effect of graded dietary GP on laying performance, yolk quality, shell ultrastructure, and egg shelf-life in laying hens. Considering this, it is important to examine the maximum dietary inclusion level of GP to optimize the laying rate, egg quality, antioxidant ability, and health status of layers.

In the current trial, the BW and BW gain of laying hens fed GP, particularly the GP6% and GP9% groups, were increased compared to the control. Similarly, egg production percent, egg weight, and egg mass were also enhanced due to adding GP to the diets of laying hens. Indeed, these enhancements were obvious after 4 weeks of feeding GP diets. Romero et al. [11], Reis et al. [18], and Kara et al. [38] recorded that dietary inclusion of GP at a level of 2% to 6% did not influence the laying rate. Reports on the dietary supplementation of GP in layers are quite scant. The inclusion of GP at 6% and 9% in the diets of laying hens enhanced egg production and egg weight in the current study, which can contribute to the enhancive effect of GP on intestinal probiotic microbiota [39]. Furthermore, the vitellogenin properties of quercetin may have been attributed to the enhanced egg production, egg weight, and egg mass noticed in the current trial. Flavonoids, such as quercetin (a phytoestrogen constituent), induce agonistic and antagonistic effects based on the internal estrogen level [40,41]. Estradiol passively assists the release and function of other reproductive hormones, including FSH, in mature hens [40,41,42]. Thus, the flavonoids present in GP may exhibit estrogenic and vitellogenin activities to improve the laying rate [40,41]. Our results are partially in harmony with Kara et al. [38], who observed that supplementing laying hens’ diets with 40 to 60 g of GP/kg of feed increased egg weight without affecting egg production. It is also essential to highlight that the use of GP flour by up to 3% in the diet of laying hens increased egg production, egg weight, and egg mass compared to the control. The inconsistent results may be attributed to different inclusion levels, grape species, diet composition, age, GP polyphenol content, and the length of the trial.

Hens fed the GP_6%_ and GP_9%_ diets had lower FI during the trial period (from week 35 to week 43 of age), but FCR was improved in all the GP-fed groups compared to the control. Considering that the dietary inclusion of GP significantly improved egg production, the reduction in FI caused an improvement in the FCR of hens fed the GP diets. These findings may indicate that feed nutrients were utilized more efficiently, as observed from the enhanced FCR. On the other hand, the reduced feed consumption may be attributed to the low acceptance of GP by hens, the elevated dietary crude fiber, NDF, and ADF contents, and anti-nutritional constituents, such as tannins and pectin. The GP diets had greater crude fiber contents (3.37%, 3.81%, and 4.25% for GP_3%_, GP_6%_, and GP_9%_, respectively) than the control diet (2.90%). However, additional research is needed to reveal the GP component(s) that may be accountable for the low acceptance of GP by hens. In quails, Silici et al. [43] observed an improvement in the feed conversion efficiency of birds fed grape seeds. Consistent with our findings, Romero et al. [11] and Kara et al. [38] showed that supplementing layers’ diets with GP decreased FI while improving FCR. Adding GP over 6% may decrease FI [44] and fat digestion [41] in broiler chickens. Overall, these results provide additional provision for the use of GP by up to 9% in the diet of laying hens without inducing detrimental effects on laying performance.

Eggs obtained from laying hens fed with GP diets linearly improved Haugh units, yolk color, albumen index, and yolk index with increased GP levels compared to the control, which can be a positive effect of supplementation, since these parameters reflect internal egg quality [18,45]. Little information exists about the impact of supplementing grape by-products on yolk color and Haugh units in layers. Our findings are in harmony with Romero et al. [11], who observed that supplementing laying hens’ diets with GP increased egg yolk color score and Haugh units. Similarly, Fróes et al. [46] reported a positive quadratic correlation between yolk coloration and dietary GP concentrations. Moreover, Haugh units were linearly improved by the addition of grape seeds and grape seed extract [19]. Amevor et al. [41] showed that dietary quercetin supplementation enhanced egg quality traits, including yolk index, albumen index, yolk color, Haugh unit, and eggshell thickness. Grapes are considered a natural source of β-carotene and lutein [44], which are frequently used as pigmenting ingredients in Europe to accomplish the most favorable yolk pigmentation anticipated by people [31]. Pigment deposition in egg yolk is determined by its dietary levels and the capability of the hen to digest, absorb, and metabolize it [46]. The improvement in the Haugh unit and yolk color score can be attributed to the flavonoid content of GP. Therefore, the dietary inclusion of GP could be attributed to somewhat reducing the dietary supplementation of synthetic pigments [11,46].

The antioxidant effects of GP, grape seed, and their extracts have been the topic of research, with the emphasis that these products have a high possibility of improving the shelf-life of animal products by mitigating lipid peroxidation [11,13,14,38,45,47,48,49]. In the present study, it was noticed that the dietary inclusion of GP by up to 90 g/kg mitigated lipid oxidation and improved the antioxidant capacity in serum, freshly laid eggs, and stored eggs. The serum and egg yolk of the GP treatments showed a lower MDA concentration and greater GPx levels than those of the control. Similarly, a reduction in lipid oxidation and enhanced antioxidant capacity were shown in the plasma and egg yolk of laying hens fed with GP [11,18,38]. It was reported that dietary resveratrol linearly decreased the egg yolk MDA concentration of quail-fed diets containing this polyphenol, which is found in red grapes and has antioxidant activity [48]. The antioxidant properties of GP and grape seed may be attributed to the phenolic compounds, which are able to scavenge free radicals, form complexes with metal ions, and hinder or lessen the formation of singlet oxygen [49,50,51]. It was revealed that GP supplementation by up to 1% of the diet was capable of lessening lipid peroxidation in the breast meat of broiler chickens [52]. It is suggested that anthocyanins found in GP may be considered potent antioxidants against lipid peroxidation in tissues by reducing free radicals [51].

The egg yolk fatty acid composition was altered in response to the dietary addition of GP. Specifically, the dietary inclusion of GP at 90 g/kg induced a reduction in the proportions of SFA, while the proportions of MUFA and PUFA improved. The GP9% egg yolk prompted the greatest level of n-3 FA and the lowest n-6 to n-3 FA ratio. Similarly, Romero et al. [11] observed that dietary inclusion of GP at 60 g/kg caused a reduction in the SFA and MUFA contents, but the PUFA content was enhanced. Dietary supplementation of 20 g/kg GP resulted in an increase in the proportion of egg yolk α-linolenic acid and a decrease in that of oleic acid and elaidic acid in quails [52]. Additionally, feeding laying hens a diet containing 30 g/kg of grape seed meal permitted a decrease in yolk contents of SFA and MUFA, while it boosted the content of PUFA [53]. Reis et al. [18] showed that dietary supplementation with GP flour by up to 3% did not affect SFA nor PUFA in egg yolk, but it increased yolk MUFA concentration. These modifications in the egg yolk FA profile of the current study could switch traditional eggs into functional foods since the obtained FA profile could prompt health benefits in humans. These changes in the egg yolk FA profile are desirable, and they can be related to the mitigation of lipid peroxidation and enhanced antioxidant capacity in the egg since lipid oxidation affects the FA profile [18]. Indeed, laying hens directly deposit the ingested dietary lipids into the egg yolk [54]. Thus, the alteration in the yolk FA proportions of laying hens fed diets including GP can be attributed to the different FA provided by GP [11,18], and it may be an associated effect with the dietary incremental levels of vegetable oil. In the research by Çelik et al. [55], the egg yolk FA profile was modified in laying hens that received grape seed oil rather than flaxseed oil. Further research is needed to highlight the mechanism of action beneath this modification in the yolk FA profile in response to dietary GP.

The improved strength of eggshells is an advantageous trait that has economic value in the commercial laying sector. It was observed that shell weight, thickness, and breaking strength were linearly improved in response to the increased dietary levels of GP. The GP9% group had the greatest values for shell weight, thickness, and breaking strength. In support of our findings, we performed scanning electron microscopy of the eggshell, and we found that the incremental dietary level of GP increased the thickness of the palisade layer but decreased both the mammillary layer and mammillary knob width compared to the control. To our knowledge, this is the first report on the effect of feeding GP on the eggshell ultrastructure. Previous studies have demonstrated that eggshell ultrastructure is an important determinant of eggshell quality [56,57]. The enhanced eggshell strength in the current trial because of feeding GP was mainly attributed to lower mammillary thickness as well as increased effective thickness and reduced width of mammillary knobs [35,58]. It was stated that the thickness of the palisade layer and the thickness and width of knobs in the mammillary layer have key roles in breaking strength [35,56,57]. The increased eggshell thickness and strength were mainly a result of the increased effective thickness due to improved calcium utilization. Previous studies have reported that flavonoids enhance eggshell thickness through the regulation of calcium metabolism through their estrogen-like effects [40,59]. However, further studies are required to confirm this mechanism of action. There is currently no research recorded on the effects of GP on the eggshell strength and ultrastructure to compare with the findings recorded herein. This study improved the tibia-breaking strength and ash content in the GP-fed laying hens compared to the control. It seems that polyphenols in GP may enhance calcium absorption and utilization, resulting in an improvement in tibia health. It has been reported that feeding grape products (rich in polyphenols) to ovariectomized rats improved calcium utilization and diminished bone turnover, causing an enhancement in bone health (greater bone calcium retention, cortical thickness, and breaking strength) [55]. Moreover, Hassan et al. [60] observed a strong relationship between an increase in osteocalcin and a rise in blood calcium after the consumption of quercetin, which may indicate an adjustment in bone mineralization [61].

Serum biochemistry is a reliable, practical approach commonly applied to monitor any alteration in response to nutrition or diseases in poultry. In this study, the dietary inclusion of GP decreased serum levels of liver enzymes (ALT and AST) within normal values, demonstrating that feeding GP did not detrimentally affect the hepatic function of hens. Laying hens on GP diets had lower serum cholesterol, triglycerides, and LDL but higher HDL compared to controls. In line with our findings, Khodayari and Shahriar [62] recorded a decrease in plasma cholesterol and triglyceride concentrations in broiler chickens fed diets with Red GP. In contrast, Kara et al. [38] and Reis et al. [18] did not observe any alteration in blood cholesterol levels in birds fed diets containing GP. The current trial obviously indicated that GP reduced yolk cholesterol concentrations. Sun et al. [39] showed that egg yolk cholesterol content was significantly decreased by diets supplemented with grape seed extract. It is well documented that eggs are an excellent source of protein and beneficial nutrients for humans [5,6,7]. However, the elevated cholesterol content is a main limiting factor for the provision of yolk because higher consumption of cholesterol was associated with an increased risk of coronary heart disease [63]. In this study, the decrease in serum and yolk cholesterol contents could be explained by the reduced absorption or synthesis of cholesterol in the intestinal tract and the enhanced bile acid excretion [39]. Polyphenols from grape seeds have been shown to inhibit HMG-CoA synthesis, the key enzyme in cholesterol synthesis, in hens and thus reduce blood and egg cholesterol [39]. Furthermore, the fiber in GP was expected to reduce blood cholesterol levels via the absorption of bile acids and different lipids [13].

## 5. Conclusions

Modification of laying hens’ diets with GP by up to 90 g/kg did not induce any detrimental effects on laying performance or egg quality traits; however, it enriched egg yolk with beneficial FA such as n-3 FA, reduced yolk cholesterol level, improved yolk color and Haugh units, and extended the egg shelf-life during storage. Moreover, the dietary inclusion of GP increased shell thickness and strength by increasing the thickness of the palisade layer while decreasing both the mammillary layer and mammillary knob width. To sum up, the inclusion of GP in the diet of laying hens by up to 90 g/kg improved laying performance, yolk quality, antioxidant status, shell quality, and shell ultrastructure without affecting laying hen health status.

## Figures and Tables

**Table 1:** The proximate analysis, total phenolic, total flavonoid, phenolic acids, and FA profile of GP.

Item	GP
metabolizable energy, kcal/kg	1625
Crude protein, %	8.94
calcium, %	3.05
Available phosphorus, %	0.22
Crude fiber, %	22.4
Acid detregent fiber, %	24.64
Neutral detergent fiber, %	36.82
Total phenolic, mg GAE/g DW	28.63
Total flavonoid, mg CE/g DW	8.42
Phenolic acids, mg/100 g DW	
Gallic acid	10.83
Ferulic acid	0.19
Chlorogenic acid	0.14
Caffeic acid	0.22
Cinnamic acid	0.69
hesperidin	0.09
Quercetin	55.02
Catechin	3.98
Fatty acid (FA) composition, %	
Saturated FA	15.53
Monounsaturated FA	19.82
Polyunsaturated FA	64.65
n-3 FA	1.78
n-6 FA	62.85

**Table 2:** Ingredients and chemical composition of the experimental diets.

Item	Control	GP_3%_	GP_6%_	GP_9%_
Ingredients, g/kg as Fed				
Corn	563.5	547.5	530	518
SBM, 44% CP	260	262	266	269
Wheat bran	75	60	41	21.4
Grape pomace	0.0	30	60	90
Vegetable oil	3	4.7	9	10
Dicalcium phosphate	15.5	16	16	16.6
Limestone	76	72.8	71	68
Premix	4	4	4	4
Common salt	3	3	3	3
Total	1000	1000	1000	1000
Nutrient composition, calculated				
ME, MJ/kg	11.41	11.40	11.41	11.41
CP, %	17.13	17.12	17.12	17.12
CF, %	2.90	3.37	3.81	4.25
Ca, %	3.33	3.31	3.33	3.32
AP, %	0.42	0.42	0.42	0.43
Lysine, %	0.90	0.90	0.90	0.90
Methionine, %	0.49	0.49	0.49	0.49

Vitamin–mineral premix provided the following per kg of diet: vit. A, 12,500 IU; vit. D, 3620 IU; vit. E, 15.4 IU; vit. K, 3.38 mg; riboflavin, 6.10 mg; niacin, 46.0 mg; vitamin B12, 20.1 µg; pantothenic acid, 14 mg; folic acid, 0.21 mg; Fe, 40.4 mg; Zn, 40 mg; Mn, 100 mg; Cu, 10 mg; I, 0.9 mg; Se, 0.21 mg; Co, 0.1 mg.

**Table 3:** Effect of experimental diets on production performance of laying hens (from 35-week-old to 43-week-old).

Items	Treatments					Significance, *p*-Value			
	Control	GP_3%_	GP_6%_	GP_9%_	SEM	Control vs. GP	L	Q	C
Initial BW, g	1819.82	1821.39	1829.62	1821.49	29.98	0.98			
Final BW, g	1903.53	1910.99	1913.69	1905.84	32.89	0.51	0.61	0.68	0.69
BW gain, g	83.71	89.60	84.07	84.35	18.89	0.76	0.82	0.62	0.84
Egg production, %									
W35–W39	88.10 ^b^	91.56 ^ab^	94.96 ^a^	92.69 ^ab^	2.62	0.04	0.05	0.14	0.51
W39–W43	83.23 ^b^	86.36 ^ab^	87.69 ^ab^	90.20 ^a^	2.32	0.02	0.008	0.91	0.80
W35–W43	85.67 ^b^	88.96 ^ab^	91.32 ^a^	91.45 ^a^	1.73	0.007	0.002	0.35	0.86
Egg weight, g									
W35–W39	57.75 ^b^	62.17 ^a^	62.55 ^a^	65.22 ^a^	1.53	0.002	˂0.001	0.43	0.21
W39–W43	63.39 ^b^	64.90 ^ab^	65.48 ^ab^	67.35 ^a^	0.87	0.003	˂0.001	0.77	0.43
W35–W43	60.57 ^c^	63.53 ^b^	64.01 ^ab^	66.29 ^a^	0.83	<0.001	˂0.001	0.57	0.12
Egg mass									
W35–W39	50.84 ^b^	56.85 ^a^	59.45 ^a^	60.47 ^a^	2.10	0.001	˂0.001	0.11	0.78
W39–W43	52.77 ^c^	56.04 ^bc^	57.44 ^ab^	60.75 ^a^	1.76	0.001	˂0.001	0.94	0.61
W35–W43	51.88 ^c^	56.49 ^b^	58.46 ^ab^	60.62 ^a^	1.18	<0.001	˂0.001	0.16	0.58
Feed intake, g/hen/d									
W35–W39	118.63 ^a^	117.96 ^ab^	117.01 ^b^	117.86 ^ab^	0.50	0.04	0.06	0.05	0.20
W39–W43	121.64 ^a^	120.16 ^ab^	118.87 ^b^	119.12 ^b^	0.60	0.001	˂0.001	0.09	0.28
W35–W43	120.14 ^a^	119.06 ^ab^	117.94 ^b^	118.49 ^b^	0.42	0.001	˂0.001	0.01	0.21
FCR, g feed/g egg mass									
W35–W39	2.34 ^a^	2.08 ^b^	1.98 ^b^	1.95 ^b^	0.07	˂0.001	˂0.001	0.04	0.71
W39–W43	2.31 ^a^	2.15 ^b^	2.07 ^bc^	1.97 ^c^	0.06	0.001	˂0.001	0.57	0.55
W35–W43	2.32 ^a^	2.11 ^b^	2.02 ^c^	1.96 ^c^	0.04	˂0.001	˂0.001	0.03	0.51

^abc^ Means with different letters within each measurement varied at *p* < 0.05; SEM = Standard error of the mean. The dietary treatments were a control group (no GP) and experimental diets containing 30, 60, or 90 g GP/kg feed (GP_3%_, GP_6%_, and GP_9%_, respectively). FCR, feed conversion ratio.

**Table 4:** Effect of experimental diets on egg yolk and albumen quality of laying hens (43-week-old).

Items	Treatments					Significance, *p*-Value			
	Control	GP_3%_	GP_6%_	GP_9%_	SEM	Control vs. GP	L	Q	C
Yolk quality									
Yolk weight, %	23.31 ^a^	20.70 ^b^	21.42 ^ab^	19.34 ^b^	0.732	0.001	˂0.001	0.61	0.02
Yolk index	65.66 ^b^	69.98 ^a^	69.96 ^a^	70.43 ^a^	0.384	˂0.001	˂0.001	˂0.001	0.002
Yolk color score	5.99 ^b^	7.55 ^a^	7.59 ^a^	7.92 ^a^	0.204	˂0.001	˂0.001	0.003	0.02
pH	6.25	6.30	6.35	6.34	0.250	0.48	0.38	0.25	0.95
**Albumen quality**									
Albumen weight, %	58.26 ^b^	59.61 ^a^	59.38 ^ab^	60.28 ^a^	0.41	0.003	0.001	0.45	0.06
Albumen index	9.48 ^b^	10.98 ^a^	11.07 ^a^	11.84 ^a^	0.37	˂0.001	˂0.001	0.19	0.10
Haugh unit	80.36 ^b^	81.33 ^ab^	81.87 ^ab^	82.62 ^a^	0.57	0.01	0.002	0.78	0.72
pH	7.99	8.16	8.10	8.17	0.430	0.67	0.76	0.36	0.80

^abc^ Means with different letters within each measurement varied at *p* < 0.05; SEM = Standard error of the mean. The dietary treatments were a control group (no GP) and experimental diets containing 30, 60, or 90 g GP/kg feed (GP_3%_, GP_6%_, and GP_9%_, respectively).

**Table 5:** Effect of experimental diets on egg yolk fatty acid (% of total fat), total cholesterol, and triglyceride contents of laying hens (43-week-old).

Items	Treatments					Significance, *p*-Value			
	Control	GP_3%_	GP_6%_	GP_9%_	SEM	Control vs. GP	L	Q	C
SFA	39.48 ^a^	36.69 ^b^	34.06 ^c^	32.36 ^d^	0.26	˂0.001	˂0.001	0.02	0.39
MUFA	37.57 ^b^	39.04 ^a^	38.13 ^b^	39.64 ^a^	0.28	˂0.001	˂0.001	0.94	˂0.001
PUFA	22.96 ^c^	24.28 ^b^	27.81 ^a^	28.01 ^a^	0.35	˂0.001	˂0.001	˂0.001	˂0.001
n-6 FA	20.98 ^c^	20.76 ^c^	22.57 ^a^	21.62 ^b^	0.36	˂0.001	˂0.001	0.001	˂0.001
n-3 FA	1.98 ^d^	3.52 ^c^	5.25 ^b^	6.40 ^a^	0.07	˂0.001	˂0.001	0.004	0.01
n-6:n-3	1.72 ^a^	1.51 ^b^	1.22 ^c^	1.16 ^d^	0.01	˂0.001	˂0.001	˂0.001	˂0.001
SFA:PUFA	1.05 ^a^	0.94 ^b^	0.89 ^c^	0.82 ^d^	0.01	˂0.001	˂0.001	0.08	0.04
SFA:MUFA	10.63 ^a^	5.90 ^b^	4.30 ^c^	3.38 ^d^	0.13	˂0.001	˂0.001	˂0.001	˂0.001
Total cholesterol, mg/g yolk	17.70 ^a^	16.68 ^a^	14.45 ^b^	11.25 ^c^	0.179	˂0.001	˂0.001	˂0.001	0.35
Triglycerides, mg/g yolk	199.67 ^a^	173.66 ^b^	125.43 ^c^	115.75 ^d^	1.68	˂0.001	˂0.001	˂0.001	˂0.001

^abcd^ Means with different letters within each measurement varied at *p* < 0.05; SEM = Standard error of the mean. The dietary treatments were a control group (no GP) and experimental diets containing 30, 60, or 90 g GP/kg feed (GP_3%_, GP_6%_, and GP_9%_, respectively).

**Table 6:** Effect of experimental diets on egg yolk antioxidant and lipid peroxidation capacity during storage.

Items	Treatments					Significance, *p*-Value			
	Control	GP_3%_	GP_6%_	GP_9%_	SEM	Control vs. GP	L	Q	C
**GPx, mg/g yolk**									
Freshly laid eggs	4.10 ^c^	4.21 ^c^	5.70 ^b^	6.57 ^a^	0.207	˂0.001	˂0.001	0.03	0.02
Stored eggs, 25 days	2.50 ^b^	3.11 ^ab^	3.85 ^a^	3.81 ^a^	0.317	0.008	0.002	0.18	0.39
Stored eggs, 45 days	2.01 ^c^	2.29 ^bc^	2.88 ^ab^	3.27 ^a^	0.256	0.005	0.001	0.75	0.55
**MDA, mg/g yolk**									
Freshly laid eggs	0.125	0.140	0.140	0.135	0.005	0.05	0.09	0.02	0.55
Stored eggs, 25 days	0.195 ^a^	0.185 ^a^	0.145 ^b^	0.140 ^b^	0.008	˂0.001	˂0.001	0.67	0.03
Stored eggs, 45 days	0.215 ^a^	0.170 ^b^	0.120 ^c^	0.121 ^c^	0.008	˂0.001	˂0.001	0.005	0.07

^abc^ Means with different letters within each measurement varied at *p* < 0.05; SEM = Standard error of the mean. The dietary treatments were a control group (no GP) and experimental diets containing 30, 60, or 90 g GP/kg feed (GP_3%_, GP_6%_, and GP_9%_, respectively). GPx, glutathione peroxidase; MDA, malondialdehyde.

**Table 7:** Effect of experimental diets on tibial physical morphology and chemical composition of laying hens (43-week-old).

Items	Treatments					Significance, *p*-Value			
	Control	GP_3%_	GP_6%_	GP_9%_	SEM	Control vs. GP	L	Q	C
Tibia weight, g	7.60	7.96	7.72	7.99	0.19	0.19	0.15	0.73	0.10
Tibia length, mm	119.81	120.38	119.82	120.28	0.50	0.25	0.38	0.43	0.53
Tibia width, mm	6.18	6.17	6.01	6.22	0.16	0.53	0.89	0.31	0.30
Tibia-breaking strength, N	139.41 ^c^	155.01 ^b^	171.14 ^a^	170.50 ^a^	1.71	˂0.001	˂0.001	˂0.001	0.01
Tibia dry matter, %	64.21	63.05	62.79	63.35	1.40	0.76	0.54	0.41	0.98
Tibia ash, %	51.56 ^b^	53.04 ^ab^	54.11 ^ab^	54.41 ^a^	0.8	0.03	0.005	0.33	0.89

^abc^ Means with different letters within each measurement varied at *p* < 0.05; SEM = Standard error of the mean. The dietary treatments were a control group (no GP) and experimental diets containing 30, 60, or 90 g GP/kg feed (GP_3%_, GP_6%_, and GP_9%_, respectively).

**Table 8:** Effect of experimental diets on eggshell quality and ultrastructural characteristics of laying hens at the end of the trial (43-week-old).

Items	Treatments					Significance, *p*-Value			
	Controlt	GP_3%_	GP_6%_	GP_9%_	SEM	Conrol vs. GP	L	Q	C
**Eggshell quality**									
Shell weight, %	18.62 ^b^	19.69 ^ab^	19.20 ^ab^	20.10 ^a^	0.394	0.02	0.008	0.76	0.04
Shell thickness, mm	0.390 ^c^	0.411 ^bc^	0.439 ^ab^	0.447 ^a^	0.010	˂0.001	˂0.001	0.36	0.43
Breaking strength, N	42.47 ^b^	45.64 ^ab^	45.67 ^ab^	49.27 ^a^	1.350	0.003	˂0.001	0.82	0.14
**Eggshell ultrastructure**									
Palisade layer, µm	238.15 ^b^	237.35 ^b^	239.69 ^ab^	242.70 ^a^	0.974	0.003	0.001	0.02	0.45
Mammillary layer, µm	51.25	51.43	51.29	50.62	0.369	0.207	0.12	0.14	0.85
Total thickness, µm	289.40 ^b^	288.78 ^b^	290.98 ^ab^	293.32 ^a^	1.119	0.015	0.004	0.09	0.46
Palisade layer, %	82.29 ^b^	82.19 ^b^	82.37 ^b^	82.74 ^a^	0.107	0.004	0.002	0.01	0.79
Mammillary layer, %	17.71 ^a^	17.81 ^a^	17.63 ^a^	17.26 ^b^	0.107	0.004	0.002	0.01	0.80
Mammillary knob width, µm	87.98 ^a^	88.56 ^a^	86.95 ^b^	86.82 ^b^	0.404	0.007	0.004	0.25	0.02

^abc^ Means with different letters within each measurement varied at *p* < 0.05; SEM = Standard error of the mean. The dietary treatments were a control group (no GP) and experimental diets containing 30, 60, or 90 g GP/kg feed (GP3%, GP6%, and GP9%, respectively).

**Table 9:** Effect of experimental diets on the blood biochemistry of laying hens (43-week-old).

Items	Treatments					Significance, *p*-Value			
	Con	GP_3%_	GP_6%_	GP_9%_	SEM	Con vs. GP	L	Q	C
**Protein metabolites**									
TP, g/dL	3.96 ^b^	4.18 ^b^	4.78 ^a^	4.91 ^a^	0.108	<0.001	<0.001	0.57	0.04
Albumin, g/dL	2.09 ^c^	2.32 ^b^	2.79 ^a^	2.83 ^a^	0.046	<0.001	<0.001	0.02	0.002
Globulin, g/dL	1.87	1.86	1.99	2.08	0.118	0.27	0.08	0.57	0.64
**Lipid profile**									
Cholesterol, mg/dL	158.16 ^a^	149.25 ^ab^	139.20 ^bc^	129.24 ^c^	4.752	0.002	<0.001	0.88	0.94
Triglycerides, mg/dL	80.26 ^a^	72.94 ^a^	61.21 ^b^	58.24 ^b^	3.216	<0.001	<0.001	0.37	0.23
HDL, mg/dL	26.50 ^c^	33.84 ^bc^	44.78 ^ab^	47.14 ^a^	3.520	0.001	<0.001	0.35	0.31
LDL, mg/dL	58.2 ^a^	39.65 ^b^	39.37 ^b^	31.91 ^c^	1.153	<0.001	<0.001	<0.001	<0.001
**Hepatic function**									
AST, mg/dL	26.80 ^a^	24.75 ^b^	22.57 ^bc^	23.15 ^c^	0.611	<0.001	<0.001	0.02	0.17
ALT, mg/dL	18.93 ^a^	15.59 ^bc^	16.64 ^ab^	12.99 ^c^	0.948	0.002	0.001	0.82	0.02
**Antioxidant status**									
GPx, U/mL	3.68 ^c^	3.84 ^b^	4.85 ^a^	4.82 ^a^	0.066	<0.001	<0.001	0.10	<0.001
MDA, nmol/mL	39.46 ^a^	37.65 ^b^	32.68 ^c^	30.16 ^d^	0.774	<0.001	<0.001	0.54	0.05

^abc^ Means with different letters within each measurement varied at *p* < 0.05; SEM = Standard error of the mean. The dietary treatments were a control group (no GP) and experimental diets containing 30, 60, or 90 g GP/kg feed (GP_3%_, GP_6%_, and GP_9%_, respectively).

## Data Availability

The data presented in this study are available on request from the corresponding author.
